# Le “limb body Wall complex”: à propos d'une observation assez particulière

**Published:** 2012-02-03

**Authors:** Myriam Rachad, Hikmat Chaara, Hakima Bouguern, Abdelillah Melhouf

**Affiliations:** 1 Service de gynéco-obstétrique II, CHU Hassan II, Fès, Maroc

**Keywords:** Limb body wall complex, échographie, diagnostic antenatal, Maroc

## Abstract

Le limb body wall complex (LBWC) est un syndrome poly-malformatif complexe et rare. Nous rapportons le premier cas, décrit au Maroc, de ce syndrome diagnostiqué en anténatal chez une primipare de 20 ans, par une première échographie obstétricale réalisée à 27 semaines d'aménorrhée. Après interruption de grossesse, l'examen macroscopique met en évidence un nouveau-né de sexe masculin, présentant tous les critères diagnostiques d'un LBWC, avec comme particularité, à notre observation, la coexistence de deux types d'attache placento-crânienne et placento-abdominale qui à notre connaissance n'a jamais été décrite auparavant, et peut mettre en question la division de ce syndrome en 2 phénotypes différents, liée à 2 mécanismes étio-pathogéniques distincts.

## Introduction

Le limb body wall complex (LBWC) est un syndrome poly-malformatif spécifique rare, peu connu par les obstétriciens et les échographistes jusqu′à 1987 ou elle a été décrit d'une manière plus précise par VAN Allen et al. [1]. Depuis cette date, il a donné lieu à une vingtaine de publications. Il associe des malformations graves pariétales antérieures, viscérales, encéphaliques rachidiennes et des membres [2]. Le diagnostic anténatal peut être porté par l’échographie anténatale, à condition qu'il soit reconnu. Dans ce but, nous rapportons un cas de diagnostic anténatal de ce syndrome réalisé à 28 semaines d'aménorrhée. A chez une primipare en précisant les particularités spécifiques à notre observation et nous essayons de faire un rappel sur les critères diagnostiques, les mécanismes étio-pathogéniques et le pronostic de ce syndrome pas aussi rare qu'on croyait.

## Observation

**Figure 1:**
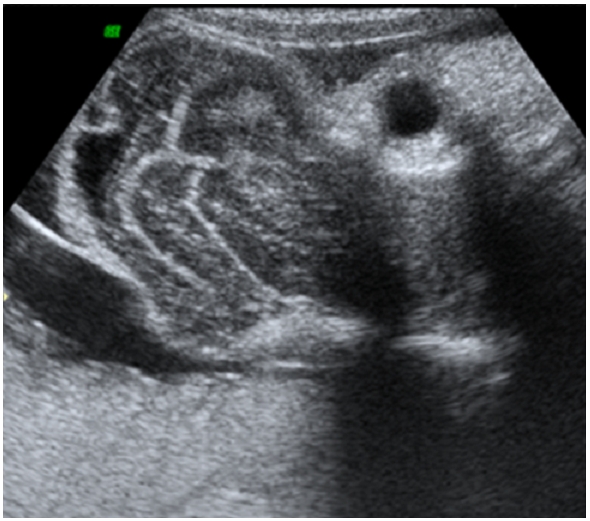
Aspect echographique de l'encephalocèle chez un foetus à 28 SA presentant un Limb Body Wall Complex

**Figure 2:**
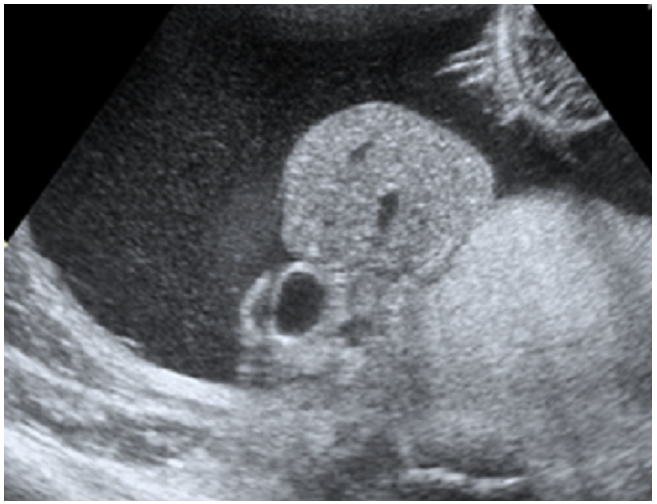
Aspect echographique de la coelosomie anterieure, avec foie, estomac et intestin qui baignent dans le liquide amniotique, chez un fœtus à 28 SA presentant un Limb Body Wall Complex

**Figure 3:**
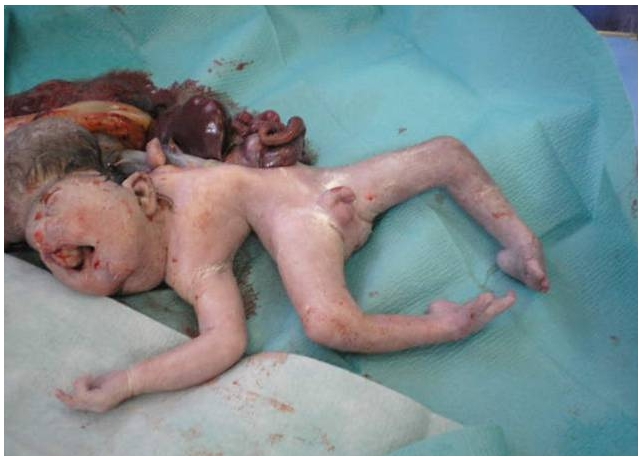
Photo du nouveau-né presentant un Limb Body Wall Complex avec syndrome poly-malformatif: encephalocele, fente complexe, coelosomie antérieure, anomalies des membres

**Figure 4:**
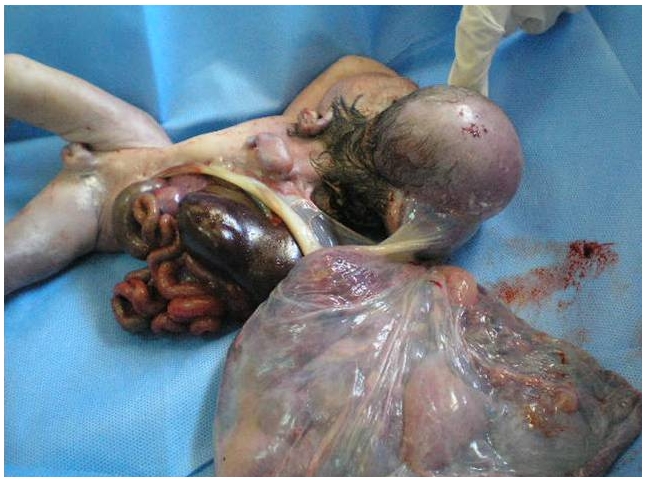
Double attache placento-cranienne et placento-abdominale chez un nouveau-né presentant un Limb Body Wall Complex

**Figure 5:**
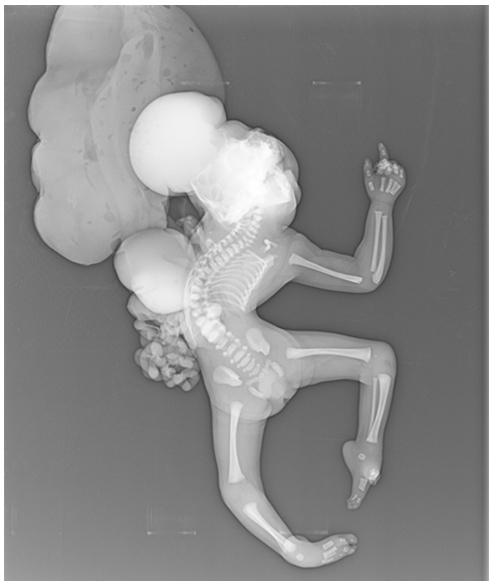
Radiographie du squelette d'un nouveau-né presentant un Limb Body Wall Complex objectivant essentiellement la scoliose importante

Mme AM, sans antécédents médicaux ou chirurgicaux particuliers. Elle est mariée depuis 1 an, sans notion de consanguinité ou de malformation foetale familiale. Primigeste, grossesse non suivi, estimée à 7 mois, sans notion de prise médicamenteuse spécifique, référée pour syndrome poly-malformatif. L'examen clinique a révélé une hauteur utérine correspondant à l’âge gestationnel avec des BCF positif et régulier.

une élève L’échographie obstétricale met en évidence une grossesse mono-foetale évolutive, liquide amitotique en quantité normale, un encéphalocèle important (Figure 1), associé a une fente labiale complexe, une coelosomie abdominale large, foie et intestin baignent dans le liquide amniotique, (Figure 2) sans individualisation de diaphragme, avec anomalies complexes des membres: absence d'individualisation du membre supérieur gauche, 2 pieds bots dont le droit avec uniquement 3 doigts. Le foetus paraissait accolé au placenta. Le diagnostic le plus probable était celui du LBWC. Une interruption médicale de grossesse, est réalisée après discussion avec les parents. L'examen macroscopique à la naissance objective un foetus de sexe masculin pesant 1800g, présentant un encéphalocèle avec fente labiale large asymétrique, une coelosomie antérieure très marqué (foie et intestin extériorisés). Un thorax étroit. Le rachis est siège d'une scoliose, le membre supérieur gauche est à l’état de bourgeon, les membres inferieurs siège de pied bots bilatérales avec anomalies quantitatives des doigts (Figure 3). Le foetus adhère au placenta par des attaches double placento-crânienne et placento-abdominales avec un cordon très court 6 cm comportant 2 artères et une veine (Figure 4). Une radiologie du squelette réalisée a objectivé une scoliose manifeste et a mis en évidence les anomalies des extrémités (Figure 5). Malheureusement, l'examen anatomopathologie à la recherche d'autres malformations viscérales n'a pas pu être réalisé car on ne dispose pas encore d'unité de foetopathologie. L'ensemble des constatations échographiques et de l'examen macroscopique ont permis cependant de retenir le diagnostic.

## Discussion

Le LBWC est un syndrome poly-malformatif complexe, dans la mesure où il n'est pas toujours reconnu comme tel, son incidence est difficile à estimer: située entre 0.21 et 0.31 pour 10000 naissances selon les auteurs [3,4]. Environ 250 cas sont relevés dans la littérature [3,5,6].

Sa physio-pathogénie est discutée est mal connue. La plupart des études ne mettent en cause aucun agent tératogène. Trois théories s'opposent. La théorie de la rupture hyper-précoce de l'amnios ou théorie « exogène » est introduite par Torpin et al en 1965 [7] et reprise par plusieurs auteurs [8,9]. Elle met en cause une interruption primaire de l'amnios induisant la formation des brides amniotiques, qui serait par un phénomène traumatique, elles-mêmes responsables des lésions foetales. Théorie la plus probable pour notre cas. Van Allen et al [1], proposent une seconde hypothèse : la théorie vasculaire ou « endogène » par infarcissement de vaisseaux embryonnaires entre 4 et 6 semaines de gestation entrainent des pertes tissulaires, une interruption dans le développement du tissu embryonnaire en raison de la nécrose hémorragique et de l'anoxie, avec déformations. Streeter en 1930 évoque un défaut primitif du disque germinal [10], concept développé particulièrement par Hartwig et al. [11,12] sous le terme de théorie de l'anomalie de fermeture embryonnaire. Ils incriminent une anomalie de développement du pédicule embryonnaire avec perturbation des plicatures caudales et latérales du disque embryonnaire à partir de 32 jours de gestation. L'ensemble conduit à une anomalie de fermeture de la paroi abdominale, à la persistance du coelome extra embryonnaire; aux anomalies du cordon, du placenta et des membres [5,13]. Cependant aucune de ces différentes théories ne semble donc expliquer l'ensemble des syndromes à elle seule.

Les critères diagnostic du LBWC sont très discutés dans la littérature. Initialement, Van Allen et al proposaient de retenir le diagnostic du LBWC lorsque deux type d'anomalies étaient présentes au moins parmi les trois suivantes [1] : exencephalie ou encéphalocèle avec fentes faciale, coelosomie thoracique et/ ou abdominale et anomalie des membres.

Plus récemment, Russo et al [5], puis Cusi et al [6] ont considéré deux phénotypes différents, selon le type d'attache placentaire. Phénotype avec attache placento-crânienne qui associe: un défaut de fermeture du tube neural dans le territoire céphalique, toujours associé avec une ou plusieurs fentes faciales complexes, une coelosomie antérieure et des brides amniotiques inconstantes. Les anomalies des membres sont facultatives et touchent surtout les membres supérieurs.

Phénotype avec attache placento-abdominale caractérisé surtout par des anomalies urogénitales complexes, des anomalies rachidiennes fréquentes. Les anomalies des sont facultatives touchent surtout les membres inférieurs. Notre observation est particulière et exceptionnel par le faite de la coexistence de deux type d'attache placento-crânienne et placento-abdominale qui à notre connaissance n'a jamais été observée auparavant.

Le diagnostic anténatal par échographie est possible dès la fin du premier trimestre, au mieux par voie vaginale, le diagnostic anténatal le plus précoce dans la littérature est réalisé à 12 SA [2]. Les anomalies les plus constamment rapportés sont la coelosomie, l'atteinte des membres, l'accolement du placenta au foetus et les anomalies rachidiennes [14–16]. Les diagnostics différentiels à évoquer seraient nombreux si l'on considérait chaque anomalie prise isolément, des difficultés diagnostiques peuvent se rencontrer en cas d'oligoamnios sévère ainsi que dans le cas d'association d'une omphalocèle ou laparosxisis avec d'autres malformation à type de dysgraphie neural ou défects cranio faciales [13,17].

Dans notre observation, notre diagnostic a principalement reposé sur l'association d'un syndrome poly-malformatif complexe avec suspicion d'accolement foeto-placentaire très caractéristique, cette suspicion d'accolement repose sur l'existence d'une proximité foeto-placentaire permanente avec limitation des mouvements foetaux.

Le pronostic du LBWC est fatal, les malformations étant malheureusement au-delà des ressources thérapeutiques, une interruption de grossesse semble acceptable dans tous les cas. Compte tenu de l'absence d'anomalies chromosomique un caryotype parait inutile.

Le risque de récurrence de l'affection était considéré nul jusqu’ à 2002 ou Leurh et all ont rapporté 2 cas de récurrence familiale pouvant faire discuter une origine génétique probable [18].

## Conclusion

Le syndrome du limb body wall complex mérite d’être mieux connu par les obstétriciens et les échographistes car cela permet à la fois de porter un diagnostic anténatal et discuter une interruption précoce de grossesse, mais surtout de rassurer les couples devant un tel syndrome poly-malformatif car le risque de récurrence est presque nul.
